# Pregnancy and delivery in a patient with aortic prosthesis for Leriche syndrome

**DOI:** 10.1590/S1516-31802003000100009

**Published:** 2003-01-02

**Authors:** Nelson Sass, Antonio Henrique Moura Poli, Maria Regina Torloni, Paula Gabriela Monteagudo Gibim, Januário de Andrade, Nilo Mitsuro Izukwa

**Keywords:** Leriche syndrome, Vascular prosthesis, Pregnancy, Labor, Delivery, Lateral recumbent position, Síndrome, Leriche, Prótese, Vascular, Gravidez, Trabalho, Parto

## Abstract

**CONTEXT:**

Leriche syndrome is a thrombotic obliteration of the bifurcation of the aorta, a rare condition that usually affects older men as a result of atherosclerosis. Women of childbearing age rarely need a vascular prosthesis (as a result of Leriche syndrome or other conditions) and there is no literature on an association between Leriche syndrome/vascular prosthesis and pregnancy/labor/delivery.

**CASE REPORT:**

A case of pregnancy and delivery in a 38-year-old patient with Leriche syndrome and an aortoiliac prosthesis is presented. The patient had no complications during pregnancy, and was admitted to the maternity hospital when close to term, to begin heparin therapy. Labor ensued spontaneously and a normal vaginal delivery occurred, resulting in a healthy infant. The authors present their considerations regarding the delivery route and the rationale for deciding in favor of vaginal childbirth.

## RESUMO

**CONTEXTO:**

A síndrome de Leriche, oclusão arterial crônica da bifurcação da aorta, é uma condição rara que acomete geralmente ho-mens idosos em decorrência de arteriosclerose. Mulheres em idade reprodutiva raramente necessitam de enxertos vasculares, associados ou não a síndrome de Leriche e não encontramos na literatura nenhuma publicação sobre síndrome de Leriche/prótese vascular e gestação/trabalho de parto/parto.

**RELATO DO CASO:**

A gestação e parto de uma paciente de 38 anos com síndrome de Leriche com prótese aorto-bilíaca são descritos. A paciente evoluiu bem durante a gestação sendo internada próximo ao termo para iniciar tratamento com heparina. O trabalho de parto iniciou-se espontaneamente, resultando em parto normal com recém-nascido saudável. Os autores apresentam suas considerações sobre a via de parto e os motivos que levaram à escolha da via vaginal.

## INTRODUCTION

Leriche syndrome is a thrombotic obliteration of the bifurcation of the aorta, a rare condition that usually affects older men due to atherosclerosis.^1^ The most frequent symptom is intermittent claudication, although other manifestations can occur. The diagnosis is based on progressive vascular insufficiency, and confirmed by angiographic studies. The ideal treatment is surgical revascularization using an aortoiliac prosthesis.^2^

Women of childbearing age rarely need a vascular prosthesis (as a result of Leriche syndrome or other conditions). Our literature search for studies on Leriche syndrome/vascular prosthesis and pregnancy/labor/delivery yielded no results. Having recently faced a case of Leriche syndrome and pregnancy, we decided to share our experience and considerations on the topic, in the hope that they could be useful to others.

## CASE REPORT

**Figure 1:**
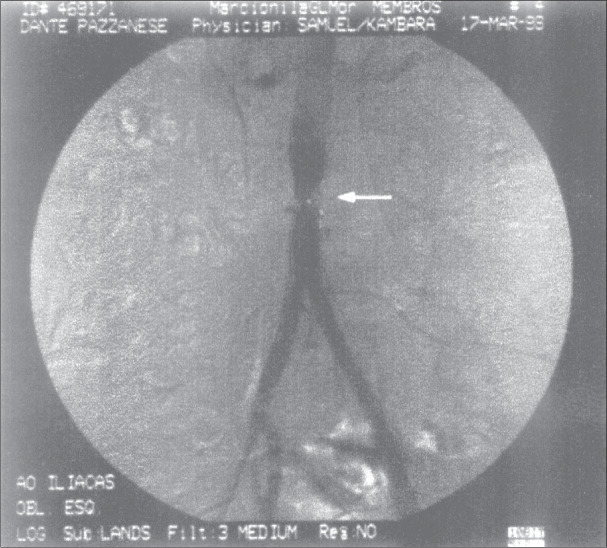
Patient's preoperative arteriography. White arrow points to the affected aortic area.

A 38-year-old asymptomatic white female was booked in for prenatal care in our Hospital in the 10^th^ week of her fourth pregnancy. Her past history was significant, with Leriche syndrome diagnosed at 36 years of age (Figure 1) and surgery (at the Instituto Dante Pazzanese) 6 months later for the insertion of an aortoiliac prosthesis (14 x 7 mm Dacron) with reimplantation of the inferior mesenteric artery. She had been smoking 10 cigarettes a day since her twenties, but quit following her diagnosis. She showed no evidence of atherosclerosis in other vessels and no previous history of heart disease, hyperviscosity or thrombotic episodes, and all her coagulation tests were normal. No other definitive cause for Leriche syndrome was identified. Her previous three pregnancies (all prior to the vascular surgery) were uneventful, resulting in term vaginal deliveries of healthy infants.

Along with her routine prenatal care (8 visits), she was also periodically being evaluated by the vascular surgeons of the Instituto Dante Pazzanese. Throughout pregnancy, she had normal peripheral pulses, blood pressure and fetal growth, and no obstetric or clinical complications. All her obstetric Doppler tests (uterine and fetal umbilical and middle cerebral arteries) were normal. She was only receiving iron tablets until the 35^th^ week, at which point she was admitted to the high-risk obstetric ward.

Following recommendations from the vascular team, she was started on subcutaneous heparin (15,000 units daily) and maintained on this medication until labor ensued, spontaneously, three weeks later. After 8 hours of regular contractions she delivered a 2,900-gram healthy boy vaginally, without episiotomy. The infant's Apgar scores were 9 and 10. No anesthesia was given during labor and the patient remained predominantly on her side until the final moments of delivery, when she was placed on her back (lithotomy). She had a normal postpartum course, resumed the use of heparin and was discharged with the infant on the 3^rd^ day.

## DISCUSSION

The occurrence of Leriche syndrome in young women is indeed rare, and smoking was the only predisposing factor identified in this patient. Heparin is not routinely recommended after vascular prosthetic surgery at the Instituto Dante Pazzanese, and this patient received the usual therapy, i.e. a short course of anti-platelet aggregating medication. However, pregnancy elevates the levels of most coagulation factors and predisposes to vascular congestion of the lower limbs, due to the compression of the pelvic veins by the gravid uterus. The risk of thrombosis is highest in the postpartum period, due to the vascular lacerations sustained during delivery. This rising risk of thrombosis prompted the vascular team to introduce heparin in the third trimester, in order to avoid this serious complication in this patient with a vascular prosthesis.

In deciding the route for delivery we had no previous experience (personal or published) on which to rely, and therefore had to deal with some doubts.

### 1. Could the fetal skull mechanically compress the prosthesis during vaginal delivery?

**Figure 2:**
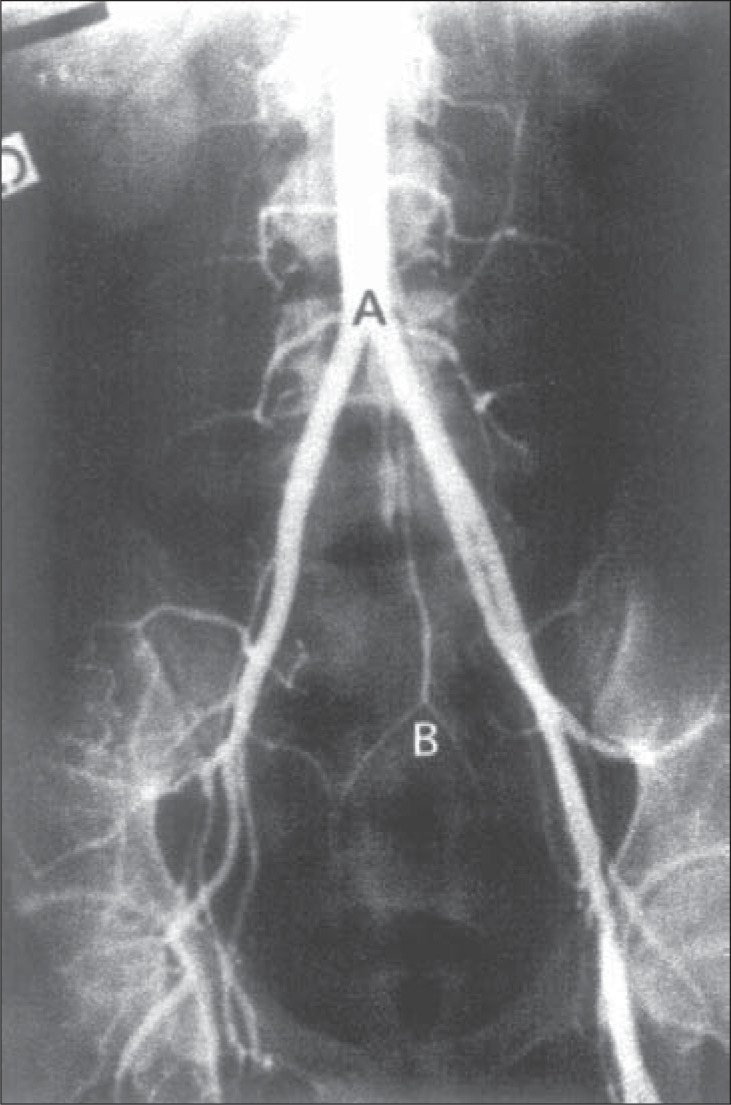
The aortic bifurcation and its anatomical relationship to the vertebrae and the bony pelvis (A: bifurcation at L4. B: promontorium).

We were concerned that the synthetic material of the prosthesis would be less resistant to external compression than a normal vessel wall, possibly collapsing and shutting off vascular flow during labor and vaginal birth. The affected area depicted in Figure 1 is situated above the bifurcation of the aorta, which occurs at the level of the fourth lumbar vertebra^3^ (Figure 2A). As can be seen on Figure 2B, the promontorium, the area of greatest contact with the fetal head during engagement, is located several centimeters below, clearly eliminating the possibility of mechanical compression of the aortic portion of the vascular prosthesis by the fetal skull. Likewise, the iliac vessels would suffer no compression during the descent of the fetal head, due to their oblique and outward course. These anatomical considerations reassured us that a vaginal delivery would not compress the prosthesis.

### 2. Would uterine contractions contribute to vascular occlusion?

During labor, along with muscular contraction of the uterus, the round ligaments are also shortened due to the existence of contractile fibers in their sheath. Emerging from the cornual and fundic portions of the uterus, the ligaments run laterally and anteriorly toward the labium major. Because of this anatomical trajectory, the contraction of the round ligaments during labor tends to pull the uterus away from the vertebrae.^4^ This movement could potentially reduce the pressure of the gravid uterus on the aorta and inferior vena cava and facilitate blood flow through the area of the vascular implant. Therefore, uterine contractions might in fact improve vascular flow through the prosthesis.

### 3. Could the patient's position during labor and vaginal delivery reduce blood flow through the prosthesis?

During prolonged dorsal decubitus, the full term gravid uterus could compress and reduce the diameter of the vascular prosthesis. We therefore encouraged the patient to maintain a lateral recumbent position during most of her labor, switching her to the dorsal lithotomy position only for the last moments of the expulsive period.

### 4. Would a cesarean section be safer?

Compared to vaginal delivery, a cesarean always entails greater risks of hemorrhage, infection and thrombosis, all potentially dangerous complications in a patient with an artificial vascular graft. These considerations helped us to decide in favor of a vaginal delivery, which ultimately occurred uneventfully.

On the basis of our experience and reflections, we believe that vaginal delivery in patients with vascular prosthesis of the aortic bifurcation is an adequate option for childbirth.
